# DA-ActNN-YOLOV5: Hybrid YOLO v5 Model with Data Augmentation and Activation of Compression Mechanism for Potato Disease Identification

**DOI:** 10.1155/2022/6114061

**Published:** 2022-09-23

**Authors:** Guowei Dai, Lin Hu, Jingchao Fan

**Affiliations:** ^1^Agricultural Information Institute of CAAS, National Agriculture Science Data Center, Beijing 100081, China; ^2^National Nanfan Research Institute (Sanya), Chinese Academy of Agricultural Sciences, Sanya 572024, China

## Abstract

To solve the problems of weak generalization of potato early and late blight recognition models in real complex scenarios, susceptibility to interference from crop varieties, colour characteristics, leaf spot shapes, disease cycles and environmental factors, and strong dependence on storage and computational resources, an improved YOLO v5 model (DA-ActNN-YOLOV5) is proposed to study potato diseases of different cycles in multiple regional scenarios. Thirteen data augmentation techniques were used to expand the data to improve model generalization and prevent overfitting; potato leaves were extracted by YOLO v5 image segmentation and labelled with LabelMe for building data samples; the component modules of the YOLO v5 network were replaced using model compression technology (ActNN) for potato disease detection when the device is low on memory. Based on this, the features extracted from all network layers are visualized, and the extraction of features from each network layer can be distinguished, from which an understanding of the feature learning behavior of the deep model can be obtained. The results show that in the scenario of multiple complex factors interacting, the identification accuracy of early and late potato blight in this study reached 99.81%. The introduced data augmentation technique improved the average accuracy by 9.22%. Compared with the uncompressed YOLO v5 model, the integrated ActNN runs more efficiently, the accuracy loss due to compressed parameters is less than 0.65%, and the time consumption does not exceed 30 min, which saves a lot of computational cost and time. In summary, this research method can accurately identify potato early and late blight in various scenarios.

## 1. Introduction

Potato is the fourth largest food crop globally after wheat, rice, and corn. At present, with potato cultivation area and total production increasing year by year [1], potato disease problems are receiving increasing attention. Among the many leaf diseases of potatoes, early and late blight are common diseases caused by a fungal infection. Potato leaves infected with early blight will wilt prematurely [2], while potato leaves infected with late blight will produce many green spots. Disease damage increases with changes in the external environment; for example, during the rainy season, when humidity is high and favours rapid fungal growth, spots can proliferate within one to two days, causing potato rhizome to become brittle and cracked [3], seriously affecting potato yields. Therefore, it is essential to establish a rapid and effective method for detecting early and late blight of potatoes.

The traditional method of leaf disease diagnosis relies on subjective human experience and is difficult to replicate and extend. With the advancement of agricultural technology, some challenging, expensive, and time-consuming methods of operation have been proposed. However, such methods require specialized equipment and operational skills that make them universally difficult to adopt [4, 5]. With the rapid development of artificial intelligence to promote precision agriculture, artificial intelligence (AI), machine learning (ML), and computer vision (CV) technologies are used for automatic crop leaf disease detection [6–8], which are time-sensitive and efficient and requires less human intervention, providing a reliable technical means for accurate detection of crop leaf diseases.

In disease identification under natural conditions, traditional computer vision feature extraction is used as a critical technical link to extract colour, texture, and shape features by HSV colour space combined with support vector machine (SVM), random forest (RF), and artificial neural network (ANN) for disease identification. However, the diversity and complexity of leaf spots under actual conditions and the susceptibility of the features to light conditions, especially the poor stability of the colour features, make this method unsatisfactory for identification [9–11]. Compared with traditional methods, convolutional neural networks (CNN) are rapidly developing, and new types of models are emerging with more substantial expressive power in feature extraction [12, 13], and VGG19, AlexNet, SqueezeNet, InceptionV3, Faster R-CNN, and ResNet50 have achieved better results in disease image detection and classification [14–16]. Tiwari et al. [17] proposed to use a pretrained VGG19 model to compare the performance of classifiers such as k-nearest neighbor (KNN), SVM, neural networks and logistic regression on the same dataset and fine-tune the target results using VGG16, VGG19 and InceptionV3 with the help of transfer learning, with a recognition accuracy of 97%, exceeding the test dataset by 8%. D. F. Wang and J. Wang [18] proposed the crop disease classification model TL-SE-ResNeXt-101, improved the deep residual network model SE-ResNeXt-101, and accelerated the convergence of the model using transfer learning techniques and data augmentation with an average accuracy of 97.99%. Yang et al. [19] proposed to train the Faster R-CNN model using a transfer learning approach and to mark out patch regions. The K-means algorithm clustered the established colour and the SIFT features and then passed them into SVM for disease classification with an average accuracy of 90.83%.

Although the above studies have made good progress in disease image classification, the related research is still only at the exploration stage of specific regional datasets, which is challenging to adapt to the current high requirements and standards of precision agriculture [20]. On the one hand, only the accuracy of scenario-specific datasets is considered, ignoring the need for CNN models to consider the impact of the scale of model parameter storage [21], resulting in the difficulty of training CNN models on resource-limited platforms. On the other hand, most methods do not evaluate their performance on unknown images [22] because of the limitations of the dataset sample, where any version of the model can be marked as good when tested on an unknown data sample. In addition, the use of classical neural network models or two-stage models [23] in training on datasets results in greater consumption of storage and computational resources due to their large number of model parameters or the need to compute the region proposal first, brings much less performance gain than one-stage [24] when integrated with other techniques, and is relatively difficult to maintain and extend later.

In this study, by introducing the one-stage YOLO v5 algorithm, balancing the depth of model width, extracting deep-level disease features, and using ActNN adaptation model activation parameter compression to minimize model storage space without compromising detection accuracy, we aim to design a target model that can identify potato early and late blights in a targeted manner while being trainable in multiple equipment environments with multiple model parameters. This work makes the following three contributions: (1) a method combining weather data augmentation with generic data augmentation is proposed for extending the data. (2) Feature visualization is used to understand the extraction of features by each network layer and to obtain global and local information about the objects of interest in each layer's convolution kernel. (3) YOLO v5 is integrated using ActNN to compress the model training memory and improve the training performance of the model in multiple device environments.

## 2. Materials and Methods

### 2.1. YOLO v5 Model Structure

**Figure 1:**
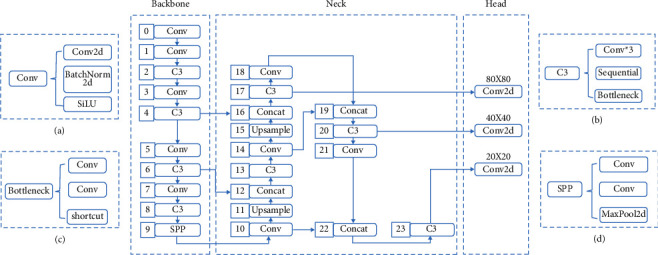
Composition and structure of the main modules (a-d) of the YOLO v5 network.

The YOLO v5 is the most representative target detection model in the one-stage series, which has high recognition accuracy and fast inference and avoids the recomputation of candidate regions in the two-stage series. The YOLO v5 architecture contains four main model structures: YOLO v5l, YOLO v5x, YOLO v5m, and YOLO v5s, and the complexity of their networks decreases in order. In order to adapt the solution to cell phones, the YOLO v5n model was later proposed, which has the same model depth and half the network width compared to the YOLO v5s, with only 1.9 MB parameters. In this study, the five models included in YOLO v5 have the same architecture (Figure 1), and YOLO v5 refers to the original network structure if not specified.

The YOLO v5 baseline architecture is mainly composed of the Backbone, Neck, and Head. Figures 1(a)–1(d) show the composition of the modules related to the baseline architecture. One of the Backbone structures is a CNN, which combines different fine-grained images and forms image features. The conv module is the basic convolution unit that runs through the architecture and performs 2D convolution, 2D regularization, and SiLU activation operations [25] on the inputs in turn. The c3 module, as the main structure for extracting features, consists of three conv with one bottleneck, and the composing structure is added to the computational graph in turn. It reduces the model size by solving the problem of large-scale repetitive gradient information and integrating gradient changes in the feature graph, reducing the model floating-point operations per second (FLOPs) and parameters and ensuring the accuracy and speed of inference. The concat module connects feature maps of different dimensions; the upsample model is used to sample feature maps. The spatial pyramid pooling (SPP) module, located at layer 9 of Backbone, is designed to increase the perceptual field of the network by transforming feature maps of any size into feature vectors of fixed size.

The Neck structure increases the flow of information through the assembly line. Low-level features can be efficiently propagated by adding feature pyramid network (FPN), a new feature with bottom-up path enhancement property. Similarly, higher-level features can be fused by adding path aggregation networks (PAN) to pass features down the line. Feature pyramids, path aggregation networks, and all other features are connected by adaptive pooling to utilise valuable information from each feature layer. The network decides from all layers which features are valid. It improves object localization accuracy by using the correct localization signals from the higher layers with solid semantic features from the lower layers.

The detection network of the Head structure consists of three detection layers, each with an input of a pixel feature map of dimensions 80 × 80, 40 × 40, and 20 × 20, for detecting image objects of various sizes. This study has three detection targets, so each detection layer outputs a 24-channel vector with three categories, one confidence level, four bounding box coordinates, and three anchor boxes ((3 + 1 + 4) × 3). The predicted bounding boxes and categories of targets in the original image are generated and labelled to enable the detection of image targets.

### 2.2. Activation Parameter Compressed ActNN

During training, deep learning models need to store model parameters, intermediate activation results, and optimizer state, which results in an exponential increase in required memory, making it difficult to train large-scale models on a GPU with limited memory. For this reason, Chen et al. [26] proposed ActNN, combining the reduced numerical accuracy BLPA of Chakrabarti and Moseley [27] and the TinyScript and nonuniform quantization strategies proposed by Fu et al. [28]. With ActNN, model parameters can be quickly compressed without negatively impacting prediction accuracy, and common CNN model structures are supported. This ActNN implements a stochastic quantized compressed version of the PyTorch neural network modules commonly used in classification, detection, and segmentation applications.

With ActNN, we implement a dynamic stochastic quantized activation neural network, which minimizes numerical precision by focusing on activation parameter contexts. As a result, training weights, activation parameters, and optimizers can be quantized to reduce numerical precision. The gradient variance convergence is affected by the quantization process, and ActNN contains a mixed accuracy quantization strategy of group quantization and fine-grained quantization, which can approximate the minimization of the gradient variance during the training process with a slight loss in accuracy in the maximum 2-bit quantization case. The model parameters are compressed using the following formula:

(1) $$\begin{array}{c} H^{\left(l\right)}=F^{\left(l\right)}\left(H^{\left(l-1\right)};\Theta^{\left(l\right)}\right), \end{array}$$

(2) $$\begin{array}{c} \nabla_{H^{\left(l-1\right)}},\nabla_{\Theta^{\left(l\right)}}=G^{\left(l\right)}\left(\nabla_{H^{\left(l\right)}},C\left(H^{\left(l-1\right)},\Theta^{\left(l\right)}\right)\right), \end{array}$$

(3) $$\begin{array}{c} \left\{\begin{array}{c} \nabla_{H^{\left(l-1\right)}}=\nabla_{H^{\left(l\right)}}\Theta^{{\left(l\right)}^{⊤}},\nabla_{\Theta^{\left(l\right)}}=H^{{\left(l-1\right)}^{⊤}}\nabla_{H^{\left(l\right)}}\\ C\left(H^{\left(l-1\right)},\Theta^{\left(l\right)}\right)=\left(H^{\left(l-1\right)},\Theta^{\left(l\right)}\right), \end{array}\right. \end{array}$$

where considering each training iteration of the *l*-layer neural network, let *X* be the input image and *Y* be the corresponding label, and one small-batch sample (*X*, *Y*) is collected from the dataset, given the input *H*^(0)^=*X* and *H*^(*l*)^ as *N* × *D*^(*l*)^, *N* is the batch size and *D*^(*l*)^ is the number of features; Θ^(*l*)^ is the parameter vector, and the forward propagation *F*^(*l*)^ contains the *N*-feature mapping *H*^(*l* − 1)^ with the model parameters Θ^(*l*)^. Given the small-batch loss function ℒ=*ℓ*(*H*^(*L*)^, *Y*) for predicting *H*^(*L*)^ and label Y, the gradient ∇ is calculated as ∇_Θ^(*l*)^_ℒ, *H*^(*l* − 1)^⊤^^ is the feature mapping transpose, and the model parameters are updated with the optimizer SGD. Since the gradient is always taken together with the loss ℒ, the gradient of the activation parameters is denoted as ∇_Θ^(*l*)^_, ∇_*H*^(*l*)^_. The process of gradient calculation can be expressed as equation (2), where the backpropagation *G*^(*l*)^ for the gradient is obtained ∇_*H*^(*l*)^_; with the context *C*(), *C*() is the information that needs to be saved in the memory for the backpropagation. In essence, this way of keeping ∇_Θ^(*l*)^_ and ∇_*H*^(*l*)^_ as full-precision gradient parameters in memory is called full-precision (FP) training. Equation (3) as a particular case, when a linear layer in *H*^(*l*)^=*H*^(*l* − 1)^Θ^(*l*)^, will carry the exact parameters of the context. ActNN to achieve 2-bit activation compression, *C*(), Θ^(*l*)^, and ∇_*H*^(*l* − 1)^_ of the *l*-layer neural network are quantized using a random quantization strategy, and the resulting lossy gradient is an unbiased estimate of the original gradient, as shown in the following equation:

(4) $$\begin{array}{c} {\hat{\nabla }}_{H^{\left(l-1\right)}},{\hat{\nabla }}_{\Theta^{\left(l\right)}}=G^{\left(l\right)}\left({\hat{\nabla }}_{H^{\left(l\right)}},\hat{C}\left(H^{\left(l-1\right)},\Theta^{\left(l\right)}\right)\right),{\hat{\nabla }}_{H^{\left(l\right)}}=\nabla_{H^{\left(l\right)}}. \end{array}$$

**Figure 2:**
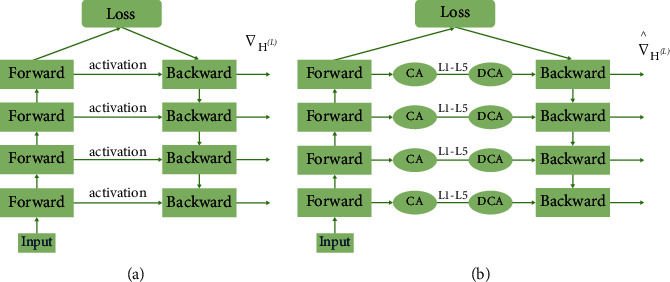
Parameter passing process (a) and ActNN compressed parameter process (b).

A hybrid accuracy quantization strategy is dynamically adjusted by ActNN at runtime to take advantage of the hardware features more effectively. As different network layers have heterogeneous characteristics, the compression algorithm reserves more bits for the most essential activation parameters. By contrast, activation parameters that have a negligible impact on the model's accuracy are compressed using a compression algorithm above the limit level, assigning an average of 2 bits per activation result, maintaining accuracy while reducing activation parameters.  Figure 2(b) shows the improvement of ActNN to the actual training process in Figure 2(a). According to Figure 2(b), ActNN defines optional compression parameters for L1 to L5, where L1 and L2 can use 4-bit per-group quantization, but L1 can use 32-bit quantization and only deals with convolutional layers; L3 to L5 use fine-grained mixed precision, swapping, and defragmentation, which acts on the activation parameters of all layers. The effects of the specific processing depend on the proportion of the original model that is processed with ActNN. The processing occurs only during training, and the detection process does not happen. In addition, as shown in (5), the compression algorithm used in L1 to L5 is a superposition of the previous compression levels. When the compression level during the training process is increased, the time it takes to decompress the activation parameters during backpropagation also increases. As a result, the training speed is decreased, even when the hardware conditions remain the same. In terms of the adjustment parameters and the data, increasing the batch size and using high-resolution images both increase the amount of time required for the compression activation (CA) parameter and the decompression activation (DCA) parameter, which in turn slows down the convergence of the model. The process of compressing and decompressing ActNN can be summarized by Algorithm 1.

(5) $$\begin{array}{c} L1⊊L2⊊L3⊊L4⊊L5. \end{array}$$

### 2.3. YOLO v5 with ActNN Integration

**Figure 3:**
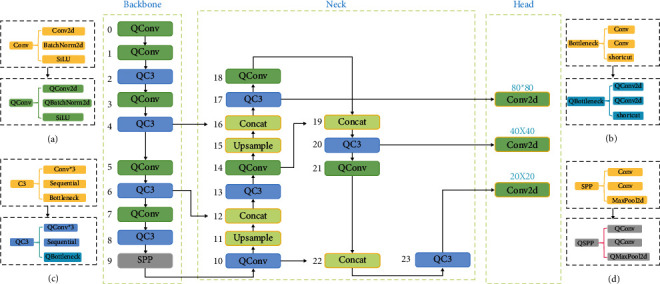
ActNN improves the model structure of YOLO v5 using the modules shown in (a-d).

In the YOLO v5 network model, the three significant structures are derived directly or indirectly from module under the nn package, an implementation based on PyTorch. The primary operations of these modules include the extraction of features, feature fusion, pooling, batch normalization (BN), and the activation function, which are essential components to measure the number of parameters of each module. With the fine-grained compression of the parameters of constituent modules, optimizations are possible from the model infrastructure without altering the model's functional structure and, consequently, without affecting the model's performance. For most modules, ActNN implements parameter compression. With Qconv and Qconvtranspose, you can create three different sizes of convolutional kernel module versions, while Qbatchnorm performs batch normalization on all three types of modules. In addition, parameter compression is also implemented for the commonly used ReLU, dropout, and maxpool2d operations. As shown in Figures 3(a)to 3(d), ActNN was used to integrate and replace some modules of the original network structure in Figure 1. Qconv, Qbottleneck, Qc3, and Qspp were selected for the replacement design of the corresponding modules. Since the parameter transfer does not affect the size of the model parameters, the upsample and concat of the original network structure are not changed; Conv2d in the Head structure outputs the detection results, during which a large number of feature parameters are passed, and these parameters are compressed using Qconv2d to obtain the improved integrated network structure DA-ActNN-YOLOV5 (Figure 3). It inherits all of the features of YOLO v5 and adds compression parameters and data augmentations. In this study, we have DA-ActNN-YOLOV5s, DA-ActNN-YOLOV5m, DA-ActNN-YOLOV5x, and DA-ActNN-YOLOV5l as the corresponding implementation of the four main YOLO v5 model structures.

### 2.4. Potato Leaf Dataset

**Figure 4:**
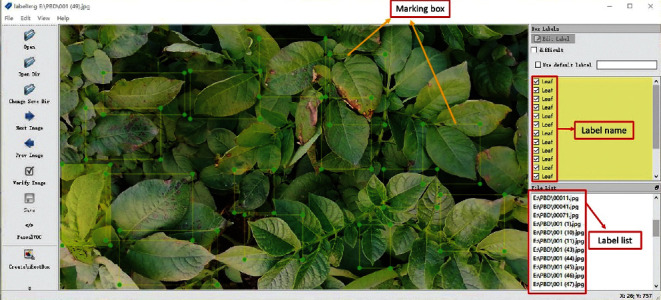
LabelMe labelling potato leaves an example.

**Figure 5:**
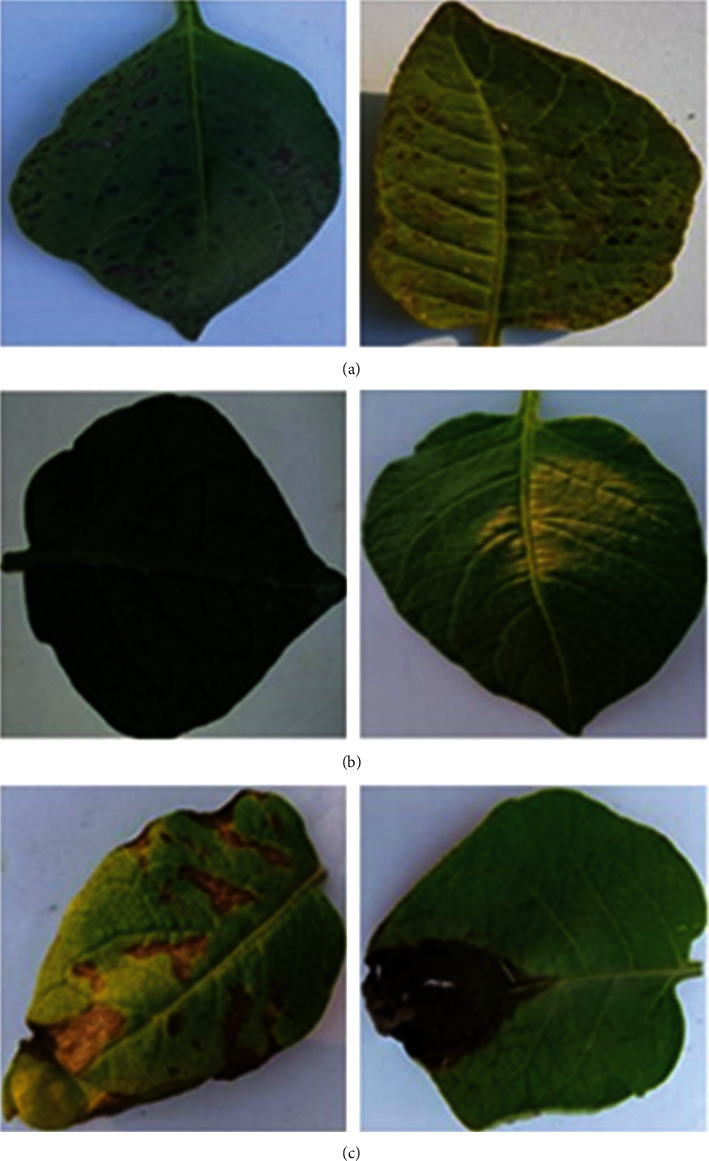
Examples of PBD-IM earlyblight (a), healthy (b), and lateblight (c).

The performance of a deep learning model depends heavily on a suitable and effective dataset. The open dataset of potato foliar diseases in existing studies is based on PlantVillage. This dataset is constructed for a specific region under specific geographical and environmental factors, and potato diseases vary in different parts of the world due to various factors such as shape, variety, and environmental factors. To improve the generalizability of the model, 1,000 early blight leaves (earlyblight), 1,000 late blight leaves (lateblight), and 152 healthy leaves (healthy) images from PlantVillage were selected as experimental subjects in this study. The selected PlantVillage dataset was trained from 0 using DA-ActNN-YOLOV5m with YOLO v5m model and tested on the PBD-IM dataset proposed in this study; as shown in Table 1, the error rate of existing PlantVillage-based models for detecting potato diseases in PBD-IM was generally high. Therefore, there is a need to reconstruct potato leaf disease datasets for the Inner Mongolia region. In this study, real-time data from the potato leaf dataset (PBD-IM) in the southeast Lingnan area of Hulunbeier City, Inner Mongolia Autonomous Region, were collected in videos and pictures. The capture distance of the cell phone camera and the digital camera was 30–40 cm, while the capture distance of the UAV was set to 1.5–3.0 m. Due to the high potato cultivation, potato varieties in the southeast Lingnan area of Hulunbeier City, Inner Mongolia Autonomous Region, were selected for this study: “Jizhang Potato No. 12,” “Hysen No. 6,” and “Huasong No. 7.” Potatoes were planted in rows 7.6 cm apart. Seeds of the crop were cultivated by digging soil pits 15.2 to 20.3 cm deep and 12.7 cm wide. To solve the problem of environmental interference in potato early and late blight identification, the dataset contains leaf samples of different disease cycles, selected for collection in fields with significant differences in leaf shape and plant growth by taking images and videos under different conditions, i.e., morning, evening, noon, cloudy, sunny versus rainy days. Healthy and infected leaves were labelled into PyTorch's YOLO v5 format and XML format using the LabelMe tool, and the labelling process is shown in Figure 4. In the segmentation and leaf extraction stages, the YOLO v5s model, which has a small number of parameters and is fast, was chosen to start training from 0. With the help of YOLO v5s model output and annotations, Python code was used to process the original labelled data and extract potato leaves to form the initial data set. To create the PBD-IM dataset, we selected 4,546 images of healthy and diseased potato leaves from the data. There are 1,828 early blight, 1,514 late blight, and 1,204 normal leaf classes, respectively, as shown in Figure 5.

**Figure 6:**
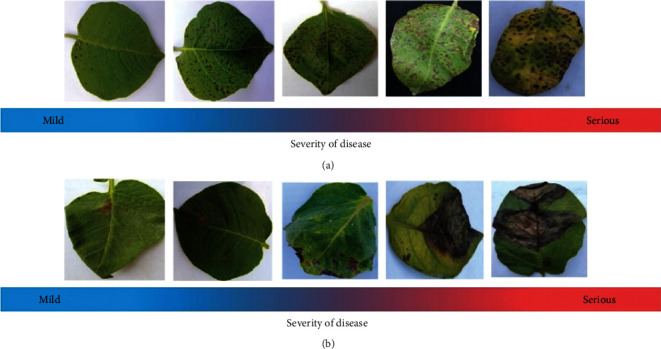
Potato early blight (a) and example of disease cycle image of late blight (b).

The growth of potato plants in the same field generally varies with the disease cycle, and to highlight the severity of potato early and late blight in the disease cycle, representative diseased leaves from PBD-IM were selected for this study (Figure 6). There is no specific quantitative definition of the degree of potato disease, and identification is made empirically by plant pathologists. In this study, the number of spots, leaf colour change, and leaf shape distortion on potato leaves were taken as the focus of attention. The cumulative values of three visual characteristics were used to discriminate the degree of disease. The focus was on these characteristics during the actual data collection.  Figure 6(a) shows the extent of early potato blight and Figure 6(b) shows the extent of late potato blight. The blue and red gradient lines in the figure represent the severity of the disease, with the left side of the line having a milder degree of disease and the right side having more severe disease. Early detection of the less severe leaves for treatment can have a positive effect on saving the whole plant. Therefore, the data creation method proposed in this study can assist in the early detection of the disease.

### 2.5. Data Preprocessing General

#### 2.5.1. Data Augmentation Methods

General data augmentation: different data augmentation techniques are applied to the training set using the image data generator of the Keras library in Python to overcome the overfitting problem and enhance the diversity of the dataset. For this reason, this article normalizes the image pixels to 0∼1, using smaller pixel parameters and the same range of pixels to reduce the computational cost without changing the properties possessed by the image itself. This study employs a combination of data augmentation techniques, using a rotation transform (Rotation_transform) to rotate the image to 25°; a width shift transform (Width_transform_range) allows the image to be shifted randomly to the right or left, choosing a width shift parameter value of 0.25. The training image is shifted up or down using a height shift range transform (Height_transform_range) with a range value of 0.25. The shear transform (Shear_transform) fixes one axis of the image and then stretches the other axis to a specific angle, called the shear angle, which is 0.23 applied in this study. The scaling range parameter is applied to perform random scaling transformations (Zoom_transform), >1.0 means expanding the image, and <1.0 is used to scale the image; therefore, a 0.2 scaling range is used to transform the image. Flip the image using a random horizontal flip (Horizontal_flip) with a parameter of 0.6. In the applied brightness transformation (Brightness_transform), 0.0 means minimum brightness and 1.0 maximum means brightness; therefore, a zoom range of 0.5∼1.0 is used. In the channel shift transformation (Channel_transform_range), a channel shift range of 0.05 is applied, while the fill mode (Fill_nearest) is used to fill in the proximity pixels.

Weather data augmentation: generic data augmentation methods are used in most studies but with limited performance improvement for object recognition errors caused by weather changes. For this purpose, we applied different weather data enhancement techniques to the training set using the image data enhancement method of the Albumentations [29] library in Python for simulating the changes in the environment induced by different weather conditions. Add random raindrops to the image via the raindrop transform (Random_rain), select a raindrop size of 1.0, type drizzle, and set the overcast parameter (brightness_coefficient) to 0.6. Due to the large temperature and humidity difference, the natural environment will produce fog. By using the fog transformation (Random_fog), fog can be added randomly to different locations of the image and blur the background; choose a parameter value of 0.1∼0.6 for fog intensity (fog_coef) and 0.1 for fog circle transparency (alpha_coef). The life activity of plants is inseparable from light. The solar light transformation (Random_sunflare) is chosen to simulate the image's natural light exposure and light adjustment. The image area where the solar flare appears (flare_roi) is determined by four parameters (x_min, y_min, x_max, y_max), x_min and y_min determine the starting coordinates, x_max and y_max determine the end coordinates; in this article, the upper left corner ((0.0,0.0,1.0,0.5)) and the upper right corner (0.9,0,1,0.5) of the image are chosen as the source of illumination. Potato plants are close to each other, and the neighboring leaves are susceptible to shading to produce the effect of shadow through the shadow transformation (Random_shadow) and to a certain extent can eliminate this effect; this study on the shadow (Shadow_roi) appeared in the region of randomization, the number of shadows in 1 ∼ 5 floating, the shadow polygon side parameters set to 4.

#### 2.5.2. Dividing the Training, Validation, and Test Sets

The PBD-IM dataset consists of training, validation, and test sets. The training dataset is used to train DA-ActNN-YOLOV5, while the validation and testing datasets evaluate the performance of the final model. This study divided the training, validation, and test datasets into 80%, 10%, and 10%, respectively. For the PBD-IM dataset, 3,638, 454, and 454 images were used for training, validation, and testing. Different data augmentation techniques were performed on the training set, i.e., rescaling, rotation, width offset, height offset, clipping range, scaling range, horizontal flip, luminance and channel offset, and proximity fill patterns, to increase diversity and enhance the dataset. It will overcome the overfitting problem and thus ensure the model's versatility.

### 2.6. Experimental Environment and Training Parameters

Experimental environment: the operating platform is Nettrix X640 G30 AI server with Ubuntu 20.04 OS, Intel® Xeon® Gold 6226R CPU, NVIDIA GeForce RTX 3090 GPU, 256 GB RAM, and 7.5 T solid-state drive. The training environment was created by MiniConda3 and configured with Python 3.8.5, PyTorch 1.10.1 with TorchVision 0.11.2 artificial neural network library. CUDA 11.1 deep neural network acceleration library was used.

Training parameter settings: DA-ActNN-YOLOV5 was selected as the PBD-IM dataset to train the network, BCELoss [30] was used for the loss function, SGD was used for the optimizer, the input image size was 640 × 640 pixels, the learning rate was initialized to 0.0042 and finally 0.15, the momentum parameter was 0.845, the weight decay was set to 0.00056, and a warm-up parameter5 is used to ensure that the model starts training with a certain prior knowledge of the data, and other parameters are kept as default. There is a correlation between the particular dataset and the convergence rate of the model training; when it seems there will no longer be any change in the model's performance or even a decrease, the training will continue, and the model will not get good convergence; at this time, this problem should be monitored, and timely intervention and the emergence of early stopping mechanism are good solutions to this problem [31]; this study integrated early stopping mechanism in DA-ActNN-YOLOV5 and set the parameter to 50.

## 3. Experimental Results and Analysis

### 3.1. Model Evaluation Metrics

To achieve the evaluation of the potato early and late blight detection model, an exhaustive analysis of the performance of the proposed method and other recent methods discussed in this study was carried out, which included various indicators such as Precision (*P*_*b*_), Recall (*R*_*b*_), F1-Score (*F*1_*b*_), Accuracy (*A*_*b*_), and Average Precision (AP). In equations (6)–(11), the formulas are separately written for each measurement indicator.

(6) $$\begin{array}{c} R_{b}=\frac{{\text{TP}}_{\text{blight}}}{{\text{TP}}_{\text{blight}}+{\text{FN}}_{\text{blight}}}, \end{array}$$

(7) $$\begin{array}{c} P_{b}=\frac{{\text{TP}}_{\text{blight}}}{{\text{TP}}_{\text{blight}}+{\text{FP}}_{\text{blight}}}, \end{array}$$

(8) $$\begin{array}{c} A_{b}=\frac{{\text{TP}}_{\text{blight}}}{{\text{TP}}_{\text{blight}}+{\text{FP}}_{\text{blight}}+{\text{FN}}_{\text{blight}}}, \end{array}$$

(9) $$\begin{array}{c} F1_{b}=\frac{2\times P_{b}\times R_{b}}{P_{b}+R_{b}}, \end{array}$$

(10) $$\begin{array}{c} AP=\frac{1}{n}\sum_{i=1}^{n}P_{b_{i}}\left(P_{b_{i}}=\frac{1}{n}P_{b_{1}}+\frac{1}{n}P_{b_{2}}+\cdots +\frac{1}{n}P_{b_{n}}\right), \end{array}$$

(11) $$\begin{array}{c} \text{mAP}=\frac{\sum_{i=1}^{Q}{\text{AP}}_{i}}{Q}. \end{array}$$

In (6)–(8),TP_blight_ (true positive), FP_blight_ (false positive), and FN_blight_ (false negative) refer to the number of positive instances that are properly recognized, the number of negative instances that are wrong recognized, and the number of positive instances mistakenly rejected, respectively. *F*1_*b*_ in (9) is a combined measure of the accuracy and recall. Usually, the accuracy and recall are mutually approximate to give a more balanced response to the model's performance. The calculation of mean average precision (mAP) depends on AP, where AP is defined as the intersection over union (IoU) threshold value of 0.5; for a class with N correctly identified samples, each correctly identified sample will correspond to a *P*_*b*_ value, and the average of the N *P*_*b*_ is taken to obtain the average accuracy of the class; see (10). mAP (IoU > 0.5) is defined as the mean value under all categories of AP, as shown in (11); this study has healthy leaves, early blight, and late blight. As the total number of detection categories, *Q* is 3, and mAP is the average cumulative value of the average accuracy of multicategory, which can overall demonstrate the comprehensive performance of the model.

### 3.2. Results and Analysis

#### 3.2.1. Analysis of Data Augmentation Results

**Figure 7:**
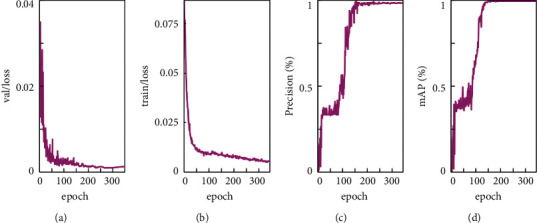
Validation loss (a), training loss, (b) verification accuracy rate, (c) and verification mAP (d).

To validate the method's superior performance on the PBD-IM dataset using the data augmentation technique applied to the training sets. This study evaluates the model using mAP, precision, and loss values, and Figure 7 depicts the complete training, validation precision, and loss in each epoch. The results show that the method achieves good recognition rates on the PBD-IM dataset using data augmentation techniques on the training set. With the intervention of the early stopping mechanism, the training stops at 350 epochs. The validation and training set's loss value curves depicted in Figures 7(a) and 7(b) drop smoothly. The loss value of the validation set reaches the lowest value at this stage at 275 epochs, and after that, all fluctuate in a very small interval. The curves depicted in Figures 7(c) and 7(d) show an extremely similar consistency, which can be specifically split into two phases. In the first phase, precision and mAP fluctuate in the 0.35–0.40 interval when the epoch is 0 to 100. In the second stage, after 60 epochs of training, the accuracy rate approaches perfect accuracy faster. After that, the accuracy rate is maintained at high accuracy until the end of training.

To evaluate the performance of the proposed data augmentation method in DA-ActNN-YOLOV5, two sets of experiments were conducted. In the first set of experiments, this study applies Table 2 ordinal number 3 data augmentation technique to the training set of the PBD-IM dataset. In Group 2 experiments, to highlight the advantages of all data augmentation techniques, this study trained DA-ActNN-YOLOV5 without using data augmentation techniques. All experimental batch sizes were 32, 100 epochs, and default learning rates. The data augmentation method used in test group 1 produced four sets of samples in each training iteration to compare the three types of data augmentation techniques applied to the PBD-IM dataset. The results are shown in Table 2. The number 1 trial used only three data augmentation technique with 97.56% accuracy; the number 2 trial used five data augmentation techniques and obtained 98.39% accuracy; the number 3 trial used nine data augmentation techniques and obtained 99.75% accuracy; experiment number 4 obtained 99.81% accuracy using 13 data augmentation techniques, with a 0.06% improvement in accuracy compared to experiment number 3, thanks to the contribution of weather data augmentation methods. The data augmentation method proposed in this study achieves high accuracy in the experimental results and generates a significant gain in DA-ActNN-YOLOV5 performance with many data samples.

Table 2 serial number 3 data augmentation techniques compared to the unused results are shown in Table 3. The experimental results showed that the proposed method was 99.38%, 99.58%, and 99.88% accurate for early blight, healthy, and late blight of potatoes. To illustrate the performance on the PlantVillage dataset, DA-ActNN-YOLOV5 was trained on the PBD-IM training set, and then the data was extracted at PlantVillage as a test set for validation, and the test results are shown in Table 4. The PBD-IM dataset showed high accuracy in detecting early and late potato diseases for PlantVillage, with a combined performance *A*_*b*_ of 95.76%, which was 8.66% higher than the test results for PlantVillage as a training set, and the performance improvement for the potato health and late blight categories was greater, with at least more than 6.04% *A*_*b*_ improvement. Doing the same test with YOLO v5m as the former, the accuracy rate was reduced by 22.75% compared to the former, proving the superiority of the proposed method over YOLO v5 in this study and also demonstrating the higher accuracy of PBD-IM compared to the PlantVillage dataset for potato disease identification. By comparison, the detection performance of the DA-ActNN-YOLOV5 network model based on the PBD-IM dataset is better.

Table 5 reviews the work related to this study. Most of the image data used to detect potatoes' early and late blight came from the PlantVillage dataset, while Rashid et al. [34] and Afzaal et al. [35] established their datasets. In this study, the shortcomings of the PlantVillage dataset were verified, and the PBD-IM dataset was established by data enhancement. By comparing the methods proposed by other researchers in Table 5, the accuracy rate of the model in this study is 0.06% and 5.81% higher than the models proposed by Rashid et al. [34] and Afzaal et al. [35], respectively. In terms of data enhancement, Chen et al. [37] used a generative adversarial network (GAN) to automatically synthesize diverse images with a 2.08% lower accuracy rate than the generic data enhancement and weather data enhancement methods proposed in this study. While comparing the remaining seven types of methods, the accuracy rate of this study is at least 3% better. Therefore, the comparative results show that the method proposed in this study achieved superior results and outperformed other research methods. In summary, this study used YOLO v5 as the base CNN model. By applying data enhancement methods to PBD-IM data samples, the original problem of high recognition error rate due to specific geographical and environmental factors was solved. Also, it reduced the differences in the degree of leaf deformation of potato early and late blight in different disease cycles in PlantVillage. Early detection of potato leaf diseases could be ensured.

#### 3.2.2. Visualization Feature

**Figure 8:**
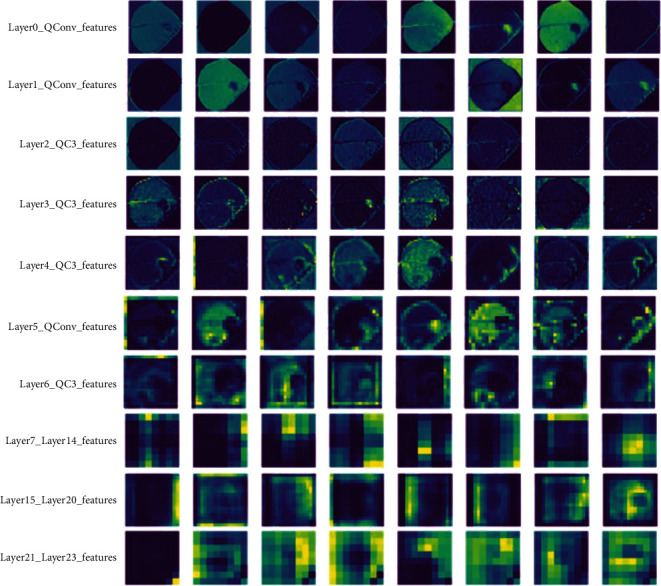
A two-dimensional 24-layer feature map was built using DA-ActNN-YOLOV5.

Visualization of neural network models can help us explore and understand the black-box learning behavior of deep models. The visualized feature map can be used as a diagnostic tool to observe the extraction of features at each network layer during model training and to diagnose potential problems with the model through a two-dimensional feature representation. By comparing filters layer by layer, we can see the effect of feature visualization of the CNN hierarchy. Each network layer based on DA-ActNN-YOLOV5 has 32 to 512 convolutional kernels, from which representative feature maps are selected as feature representations for each network layer, and Figure 8 shows the 24-layer feature visual representation learned by the model. It can be observed that the convolutional kernels in layers 0 to 1 acquire the ability to extract image edges by learning samples, and the edges of potato leaf and leaf spot images are visible. It is worth noting that different convolutional kernels do not focus on the same object; some focus more on the leaf edges, others on the edges of the disease spots, and a few focus on both leaf and disease spot edges. In the network extracted feature maps of layers 2 to 4, the convolutional kernel focuses on extracting potato leaf texture features, and the overall structure of potato leaf veins is better expressed. In the feature maps extracted by the 5- to 6-layer network, the edge and texture information becomes blurred, and the shape of the generated image changes from fine to coarse, e.g., the image representation obtained using the Gaussian blurring process. From layers 7 to 23, the feature representation becomes more complex with the increase of network layers, from which the information of edge and texture features of the leaf cannot be distinguished. Therefore, the network extracted features can be seen as a mapping process from low-to high-dimensional features, and the increase of network layers and filters makes the features more abstract.

#### 3.2.3. Analysis of ActNN Results

The training will not continue when the training model exceeds the maximum memory capacity supported by the user device. Among the network models provided by YOLO v5, YOLO v5x, YOLO v5m, and YOLO v5s were used most frequently. The three models were tested on the PBD-IM dataset, and except for YOLO v5x, which could not be trained, YOLO v5m and YOLO v5s training were able to proceed normally, but YOLO v5m was close to the device memory threshold in training and could not be trained using a deeper network structure. The usual solution to the problem of training not being possible due to limited device memory is to reduce the batch size or reduce the input image size. However, this approach has limited usefulness, and training may also not be possible when the batch size is 1. Moreover, for the problem that the device memory of the training model is close to the critical value, the effect of reducing the batch size and the input image size method is more significant. The DA-ActNN-YOLOV5 proposed in this study can use ActNN to compress the memory when out of memory (OOM) occurs in the device.

For this purpose, three sets of experiments were performed to evaluate the performance of DA-ActNN-YOLOV5. Instead of choosing DA-ActNN-YOLOV5s with fewer parameters, experiments were performed using DA-ActNN-YOLOV5l with more model parameters. Since the ActNN compression level, batch size, and input image size are different for each group of tests, the model will not get better convergence if a consistent epoch is used, so the tests do not restrict the epoch, and other settings are set concerning the base parameters to ensure that the model converges to a better state for each group of tests. All experimental groups verified 3 compression levels, containing L1, L3, and L5, and L2 and L4 were not selected, mainly because their algorithmic advantages were not obvious. In three groups of experiments, 13 data enhancement techniques were applied to the model trials, and each group was first ensured to train without ActNN. The training process only changed the batch size and image sizes, and ActNN was turned on when the device memory was insufficient. The compression level was tested three times in each group, the optimal values were recorded, and the results are shown in Table 6.

In all three sets of model tests using ActNN, the models achieved a high level of accuracy without any significant degradation in accuracy. They had the best accuracy rate for a batch size of 64. In addition, a smaller batch size corresponds to slightly smaller accuracy due to the low training sample in each iteration, and choosing a larger batch size for the same training can improve the model accuracy and make the model converge quickly. The model complexity of DA-ActNN-YOLOV5m, DA-ActNN-YOLOV5x, and DA-ActNN-YOLOV5l increases sequentially, and when the device memory is insufficient due to changing the batch size and image size, the ActNN compressed model parameters enable the model to be trained normally. In addition, the accuracy loss from compressing model parameters is less than 0.65% for the highest compression level L5 compared to the lowest compression level L1, and the additional time consumed for compressing model parameters is less than 30 min compared to the model without ActNN. In addition, higher compression levels will bring additional computation time consumption, and the compression level of ActNN should be chosen reasonably according to the actual situation.

The proportion of ActNN module replacement selected for the neural network model affects the recognition accuracy and training time to different degrees. In order to analyze the performance of the three network structures of YOLO v5 after replacing the ActNN module, the DA-ActNN-YOLOV5l model is used as an example; the batch size is set to 32, the image size is 384 pixels, and the compression parameter level is selected as L3, and the experimental results are shown in Table 7. When parameter compression is enabled, all three network structures have different degrees of accuracy loss. Among them, since the Head structure is only involved in the output of the feature map and does not involve the intermediate transfer process of the feature map, compressing the parameters of the Head structure has the most negligible impact on the accuracy, and the accuracy loss is less than 0.15%. Ten modules of the Backbone structure were replaced with ActNN modules, accounting for 47.1% of the total replacement modules. Since feature extraction is the main work of the Backbone structure and the convolutional computation is quite large, compressing the Backbone structure parameters resulted in a 1.32% decrease in accuracy. In addition, the Neck structure accounted for 38% of the total replacement modules and compressed parameters resulted in a 0.81% reduction in accuracy. In a comprehensive analysis, the number of modules involved in compressing parameters is the main reason for the decrease in accuracy and increase in training time. The increase in accuracy loss and training time is positively correlated with the increase in compression level.

## 4. Conclusion

This study proposes a potato early and late blight detection method based on the deep learning YOLO v5 framework called DA-ActNN-YOLOV5. By replacing the corresponding module of YOLO v5 with ActNN, the fine-grained compression of the activation parameters is achieved. Compared with the model with uncompressed parameters, the highest compression level results in an accuracy loss of less than 0.65%, and the time consumed to compress the model parameters is less than 30 minutes, which is an obvious advantage. When training with models of different complexity, a reasonable choice of different compression levels for the model activation parameters can accelerate the model to converge quickly with approximate original accuracy and compress the model parameters to ensure smooth training when the device memory is insufficient. The accuracy of DA-ActNN-YOLOV5 in identifying early blight, healthy leaves, and late blight of potatoes using 13 data enhancement techniques was 99.38%, 99.58%, and 99.88%, which were 5.81%, 14.41%, and 7.43% higher than without data enhancement; the average accuracy was 9.22% higher. Compared to YOLO v5, performance was improved by at least 6%, with a mAP of 99.81% (IoU ≥ 0.5) for the retained test dataset. In addition, the performance of the PBD-IM dataset outperformed PlantVillage, with an 8.66% improvement in accuracy after the DA-ActNN-YOLOV5 test and a more significant performance improvement in the identification of healthy leaves and late blight categories, with an average of 6.04% improvement in accuracy. To obtain the extraction of features by the entire network layer, we visualized the feature maps extracted by each network layer. Each layer of the feature extraction network has its division of labour, with some network layers focusing on object edges and others on object colour textures. The overall feature extraction process can be viewed as a mapping process from low-to high-dimensional features. The results show that the method proposed in this study can effectively discriminate potato early and late blight under different leaf shapes, pathogenesis cycles, and environmental factors; it solves the problem of potato early and late blight staying on specific regional data sets and realizes model training for multiple equipment environments. It can be used as a reference tool for other crop disease detection and building detection models, helping researchers accelerate the application of artificial intelligence in agriculture in other directions and meeting the requirements of precision agriculture falling in the field. In the future, this research will be expanded to include multiple disease detection on individual leaves and enhancements to disease localization and disease severity estimation—development of an IoT-based real-time monitoring system, website development, and mobile application release.

## Figures and Tables

**Algorithm 1:**
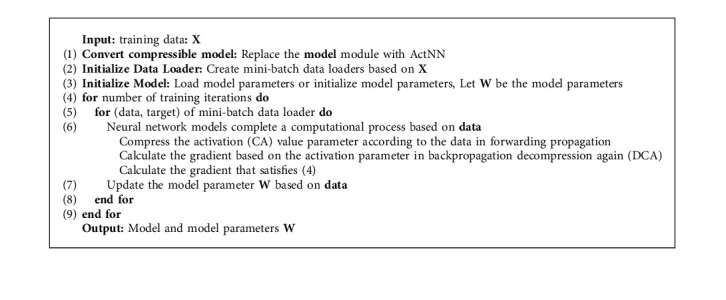
Algorithm description of ActNN compression process. The training process uses minibatch random gradient descent training.

**Table 1:** Accuracy of PlantVillage trained models in PBD-IM tests.

Model	Training set	Testing set	Precision (%)			Total images	*A*_*b*_(%)
			Earlyblight	Healthy	Lateblight		
YOLO v5m	PlantVillage	PBD-IM	94.71	9.82	26.94	900	49.79
DA-ActNN-YOLOV5m	PlantVillage	PBD-IM	96.28	89.39	75.64	900	87.10

**Table 2:** Accuracy of the DA-ActNN-YOLOV5m model using different data augmentation methods in the PBD-IM dataset.

No.	Data augmentation methods	Precision (%)			mAP (%)
		Earlyblight	Healthy	Lateblight	
1	Rotation_transform	96.72	97.56	96.43	97.56
	Width_transform_range				
	Height_transform_range				

2	Rotation_transform	98.87	98.44	97.85	98.39
	Width_transform_range				
	Height_transform_range				
	Shear_transform				
	Zoom_transform				

3	Rotation_transform	99.12	100.00	98.94	99.75
	Width_transform_range				
	Height_transform_range				
	Shear_transform				
	Zoom_transform				
	Horizontal_flip				
	Brightness_transform				
	Channel_transform_range				
	Fill_nearest				

4	Rotation_transform	99.98	100.00	99.85	99.81
	Width_transform_range				
	Height_transform_range				
	Shear_transform				
	Zoom_transform				
	Horizontal_flip				
	Brightness_transform				
	Channel_transform_range				
	Fill_nearest				
	Random_rain				
	Random_fog				
	Random_shadow				
	Shadow_roi				

**Table 3:** Test performance of the DA-ActNN-YOLOV5m model on the PBD-IM dataset using data augmentation methods.

Method	Mertics (%)	Earlyblight	Healthy	Lateblight	AP (%)
**With data augmentation**	*A*_*b*_	99.38	99.58	99.88	99.61
	*P*_*b*_	99.01	100.00	100.00	—
	*R*_*b*_	98.04	100.00	99.03	—
	*F*1_*b*_	98.05	98.06	99.09	—

**Without data augmentation**	*A*_*b*_	93.57	85.17	92.45	90.39
	*P*_*b*_	88.01	93.05	90.00	—
	*R*_*b*_	91.03	86.04	87.06	—
	*F*1_*b*_	89.06	89.00	88.04	—

**Table 4:** Accuracy of models trained in PBD-IM tested in PlantVillage.

Model	Training set	Testing set	Precision (%)			Total images	*A*_*b*_ (%)
			Earlyblight	Healthy	Lateblight		
YOLO v5m	PBD-IM	PlantVillage	92.37	56.91	69.76	500	73.01
DA-ActNN-YOLO V5m	PBD-IM	PlantVillage	97.29	94.56	95.43	500	95.76

**Table 5:** The methodology of this study compared to other research methods.

No.	References	Year	Method description	AP (%)
1	Sholihati et al. [32]	2020	VGGNet16 + VGGNet19	91.31
2	Yang et al. [19]	2020	Faster R-CNN + SIFT + K-means	90.83
3	Barman et al. [33]	2021	Self-build CNN (SBCNN)	96.98
4	D. F. Wang, and J. Wang [18]	2021	SE + ResNet50+DenseNet-121	97.99
5	Rashid et al. [34]	2021	PDDCNN + data augmentation	99.75
6	Afzaal et al. [35]	2021	GoogleNet + VGGNet + EfficientNet	94.00
7	Hou et al. [36]	2022	k-NN + SVM + ANN + RF	97.40
8	Chen et al. [37]	2022	MobileNetV2+GAN + Attention mechanism + octave	97.73
9	Mahum et al. [38]	2022	DenseNet-201 + Efficient DenseNet	97.20
10	Sharma et al. [39]	2022	Deep CNN	97.66
11	Proposed model	2022	YOLO v5+ data augmentation + ActNN (DA-ActNN-YOLOV5)	99.81

**Table 6:** mAP and training elapsed time of DA-ActNN-YOLOV5 on the PBD-IM dataset.

Model	Batch size	Image size	Level	mAP (%)	Time (h)
**DA-ActNN-YOLOV5m**	32	384	—	98.76	1.852
	64	384	—	99.86	1.579
	128	384	—	98.99	1.286
	64	512	—	99.68	1.685
	128	512	L1	OOM	—
	128	512	L3	98.66	1.663
	128	512	L5	99.12	1.681

**DA-ActNN-YOLOV5x**	16	384	—	97.23	2.869
	32	384	—	98.68	2.521
	64	384	—	99.78	2.219
	64	512	L1	OOM	—
	64	512	L3	97.99	2.674
	64	512	L5	98.39	2.683

**DA-ActNN-YOLOV5l**	8	384	—	96.89	3.853
	16	384	—	97.38	3.457
	32	384	—	98.97	3.289
	32	512	—	OOM	—
	32	512	L1	OOM	—
	32	512	L3	98.57	3.565
	32	512	L5	98.19	3.723

**Table 7:** Ablation experiments of the three main structures of ActNN replacement DA-ActNN-YOLOV5l.

No.	Compression network structure			mAP (%)	Time (h)
	Backbone	Neck	Head		
1	✓			97.65	3.375
2		✓		98.16	3.358
3			✓	98.83	3.324
4	✓		✓	97.55	3.412
5		✓	✓	98.07	3.395
6	✓	✓		97.41	3.467
7	✓	✓	✓	97.34	3.474

## Data Availability

The data that support the findings of this study are available on request from the corresponding author. The data are not publicly available due to privacy or ethical restrictions.
